# Relative burden of cancer and noncancer mortality among long-term survivors of differentiated thyroid cancer in the US

**DOI:** 10.3389/fendo.2024.1425634

**Published:** 2024-10-25

**Authors:** Yang Shi, Chengzhou Lv, Pai Liu, Yuenan Zheng, Hao Zhang, Wenwu Dong, Ping Zhang

**Affiliations:** Department of Thyroid Surgery, The First Hospital of China Medical University, Shenyang, China

**Keywords:** differentiated thyroid cancer, prognosis, competing risks model, DTC, SEER

## Abstract

**Background:**

Limited information is available regarding the relative risks of cancer-specific mortality and noncancer-specific mortality among long-term survivors with differentiated thyroid cancer (DTC).

**Methods:**

In this retrospective study, nationwide data from the Surveillance, Epidemiology, and End Results database (1992-2020) were utilized. The Accelerated Failure Time Model was applied to calculate Survival Time Ratios (TR), with the primary focus on mortality resulting from DTC. The competing risks model was employed to investigate the relative risks of various outcomes in DTC patients with a survival duration of 5 years or more.

**Results:**

In our cohort, 279 patients succumbed to DTC, while 748 died from other diseases. Notably, in DTC cohorts, noncancer-specific mortality rates were consistently higher than DTC-specific mortality rates across different age groups and genders. The risk of DTC and noncancer-specific mortality varied based on the TNM stage. With more advanced disease stages, the risk of DTC and other cancer-specific mortality gradually increased. The cumulative mortality from other cancer-specific causes was consistently the lowest.

**Conclusions:**

In long-term surviving DTC patients, noncancer-specific mortality outweighed DTC-specific mortality irrespective of age and gender. For stage I and II patients, increased attention should be directed toward noncancer-specific mortality in postoperative follow-ups. Conversely, for stage III and IV patients, greater consideration should be given to DTC-related causes of death. In addition, for stage III and IV DTC patients, the risk of death from other cancers was significantly higher than for stages I and II.

## Introduction

Thyroid cancer stands as the most prevalent endocrine malignancy, with differentiated thyroid cancer (DTC) comprising around 90% of thyroid cancer (1). According to the 2023 Cancer Data Statistics (2), thyroid cancer is the 7th most common cancer in women (3%). Enhanced prognosis resulting from early detection and treatment has led to a noteworthy 5-year disease-specific survival rate of 98% for thyroid cancer patient (3). As a result, an increasing number of cancer survivors experience extended life spans, facing a higher likelihood of encountering or succumbing to other diseases.

Despite the improved prognosis for DTC patients, existing studies lack an evaluation of the varying risks associated with distinct causes of death for long-term survivors. Notably, these studies do not differentiate between factors associated with DTC-specific and nonDTC-specific mortality. Hence, there is a critical need to investigate the relative risks of cancer-specific and noncancer-specific mortality among long-term surviving DTC patients. Given that in our study, the Kaplan-Meier (KM) estimation procedure may not be directly suitable in the presence of competing risks, we employed a competing risks model to explore the relative risks of different outcomes in DTC patients who have surpassed the 5-year survival mark. Additionally, we quantified the relative long-term risk of DTC versus nonDTC-specific mortality to aid physicians in tailoring healthcare monitoring for DTC patients with varying risk profiles. This approach aims to provide valuable information for survivor care, adapting to the unique risk characteristics of individual patients.

## Materials and methods

**Figure 1:**
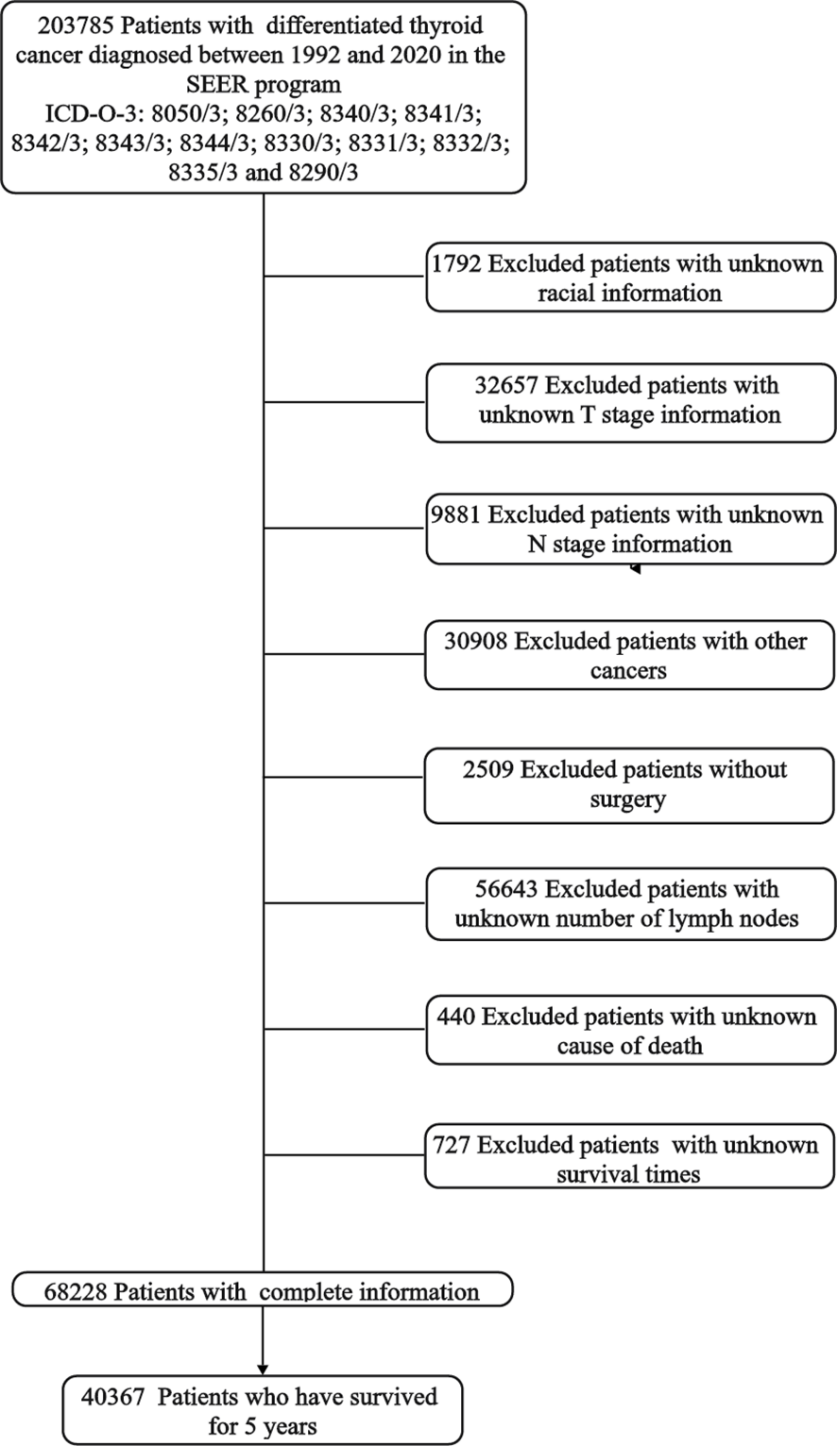
Flowchart.

This retrospective study utilized nationwide data obtained from the Surveillance, Epidemiology, and End Results (SEER) database (1992–2020) of the National Cancer Institute. The SEER database, comprising information from 12 population-based cancer registries covering approximately one-third of the U.S. population, serves as a robust source for collecting and providing incidence and survival statistics. This extensive dataset spanning 15 years is a major strength of our study, providing a large sample size that enhances our ability to detect relative risks of cancer-specific and noncancer-specific mortality. This extensive dataset spanning 15 years is a major strength of our study, providing a large sample size that enhances our ability to detect relative risks of cancer-specific and noncancer-specific mortality. To identify patients diagnosed with differentiated thyroid cancer, we employed histopathology codes based on the International Classification of Disease for Oncology (3rd edition; ICD-O-3), specifically 8050/3; 8260/3; 8340/3; 8341/3; 8342/3; 8343/3; 8344/3; 8330/3; 8331/3; 8332/3; 8335/3, and 8290/3. Our study focused on patients who received a definitive treatment for DTC and survived for at least 5 years post-diagnosis. Exclusion criteria were applied to eliminate patients with incomplete or missing tumor-related information, those lacking surgery, individuals with unknown survival times, or those with more than one type of primary cancer. Data extracted from the SEER database included age at diagnosis, sex (male or female), race (Non-Hispanic White, Non-Hispanic Black, Non-Hispanic American Indian/Alaska Native, Non-Hispanic Asian or Pacific Islander or Hispanic), tumor size (≤4cm or >4cm), number of lymph node metastases (≤5 or >5), quantity of tumors (solitary or multifocal), the 8th AJCC TNM staging system was based on tumor information provided by the database, months from diagnosis to treatment (immediate, ≤6 months, >6 months) and treatment strategy (surgery and radioiodine therapy). Surgical characteristics were categorized as lobectomy or less (LT) and total thyroidectomy or near-total (TT). The flowchart is shown in **Figure 1**. A total of 40367 DTC patients met the inclusion criteria for this study. The Institutional Review Board of the First Hospital of China Medical University deemed this study exempt from review, considering the anonymized nature of the SEER database and its accessibility after obtaining permission.

### Analyses of statistical data

Continuous variables were summarized as medians with interquartile ranges (IQRs), while categorical variables were presented as counts and proportions. In this study, the outcomes of DTC were categorized into DTC-specific mortality, and mortality attributed to other causes was defined as noncancer-specific mortality and other cancer-specific mortality. These classifications were based on the SEER cause-of-death classification variables. Given that several covariates did not meet the proportional hazards assumption, we opted for the accelerated failure time (AFT) model. Time ratios (TR) greater or less than 1 indicate increased or decreased survival time, respectively. The AFT model was employed to identify common factors affecting both types of mortality, and subsequently, the competing risk model was utilized to compare the risk of death for different causes. A two-sided test was conducted for all data, with statistical significance set at a *P* value less than 0.05. Statistical analyses were carried out using SPSS software (version 26.0; IBM, Armonk, NY, USA) and the R Language (version 4.2.3; Nashville, USA).

## Results

### Demographic and clinicopathological features

**Table 1** provides a summary of the baseline characteristics of the study population. The median age at diagnosis was 46.6 years, with 74.76% of patients being under the age of 55. DTC was more prevalent in females (78.72%) and in non-hispanic white patients (67.33%). Using a tumor size cutoff of 4 cm, 36671 patients (90.84%) had tumors ≤4 cm. Solitary tumors were observed in 98.64% of the cohort. Using a number of lymph node metastases cutoff of 5, 35184patients (87.33%) ≤5 lymph node metastases. According to the 8th AJCC TNM staging system, 35966 (89.10%), 3755 (9.30%), 370 (0.92%) and 276 (0.68%) patients were classified as stage I, II, III and IV, respectively. 53.18% of patients were treated immediately after diagnosis, and 46.08% were treated within 6 months. Total thyroidectomy or near-total procedures were performed in 37408 (92.67%) patients, while radioiodine therapy was administered to 21895 (54.24%) patients.

**Table 1:** Baseline characteristics of patients with DTC.

Characteristic	No. of patients (%)
Age, mean (SD), y	46.6 (14.3)
<55	30178 (74.76)
≥55	10189 (25.24)
Sex	
Female	31777 (78.72)
Male	8590 (21.28)
Race	
Non-Hispanic White	26994 (66.87)
Hispanic	6933 (17.17)
Non-Hispanic Asian or Pacific Islander	4488 (11.12)
Non-Hispanic Black	1717 (4.25)
Non-Hispanic American Indian/Alaska Native	235 (0.58)
Size	
≤4cm	36671 (90.84)
>4cm	3696 (9.16)
Foci	
Solitary	39818 (98.64)
Multifocal	549 (1.36)
Number of lymph node metastases	
≤5	35184 (87.16)
>5	5183 (12.84)
T	
T1a	13276 (32.89)
T1b	11305 (28.01)
T2	8429(20.88)
T3a	3328 (8.24)
T3b	2656 (6.58)
T4a	1002 (2.48)
T4b	371 (0.92)
N	
N0	24534 (60.78)
N1a	10082 (24.98)
N1b	5751 (14.25)
M	
M0	40019 (99.14)
M1	348 (0.86)
8th AJCC Stage	
I	35966 (89.10)
II	3755 (9.30)
III	370 (0.92)
IV	276 (0.68)
Months from diagnosis to treatment	
immediate	21466 (53.18)
≤6 months	18600 (46.08)
>6 months	301 (0.75)
Surgery	
Lobectomy or less	2959 (7.33)
Thyroidectomy or near total	37408 (92.67)
Radioiodine therapy	
Yes	21895 (54.24)
No	18472 (45.76)

Differentiated thyroid cancer (DTC); SD, standard deviation.

### Cause of death

In our cohort, a total of 1027 patients succumbed to various causes (**Table 2**). Among these, 505 patients passed away within the 5-8 years after diagnosis, 162 patients (32.08%) passed away due to causes unrelated to primary cancer, with diseases of the circulatory system accounting for 33.86% (162/505) of these nonDTC-specific deaths. Following circulatory system disease, other common causes of nonDTC-specific mortality included diseases of the nervous and mental system (9.31%, 47/505) and other cancer (5.35%, 27/505) (**Table 2**). At >12 years after diagnosis, there were only 25 deaths from DTC, diseases of the circulatory system (n=45, 34.09%), diseases of the respiratory system (n=19, 14.39%), and other cancer (n = 8, 6.06%).

**Table 2:** Causes of death DTC patients who had survived 5 years.

	5-8 years	9-12 years	< 12 years
Total number of deaths (n = 1027)	505	390	132
Cause of death			
DTC	162 (32.08)	92 (23.59)	25 (18.94)
Other cancer	27 (5.35)	22 (5.64)	8 (6.06)
Diseases of the circulatory system	171 (33.86)	125 (32.05)	45 (34.09)
Diseases of the respiratory system	21 (4.16)	28 (7.18)	19 (14.39)
Diseases of the nervous and mental system	47 (9.31)	42 (10.77)	12 (9.09)
Diabetes Mellitus	12 (2.38)	17 (4.36)	4 (3.03)
Diseases of the urinary system	14 (2.77)	10 (2.56)	3 (2.27)
Diseases of the digestive system	10 (1.98)	4 (1.03)	1 (0.76)
Certain infectious and parasitic diseases	5 (0.99)	15 (3.85)	3 (2.27)
Septicemia	8 (1.58)	10 (2.56)	2 (1.52)
Accidents and Adverse Effects	28 (5.54)	25 (6.41)	10 (7.58)

### Factors associated with DTC and nonDTC-specific mortality

**Figure 2:**
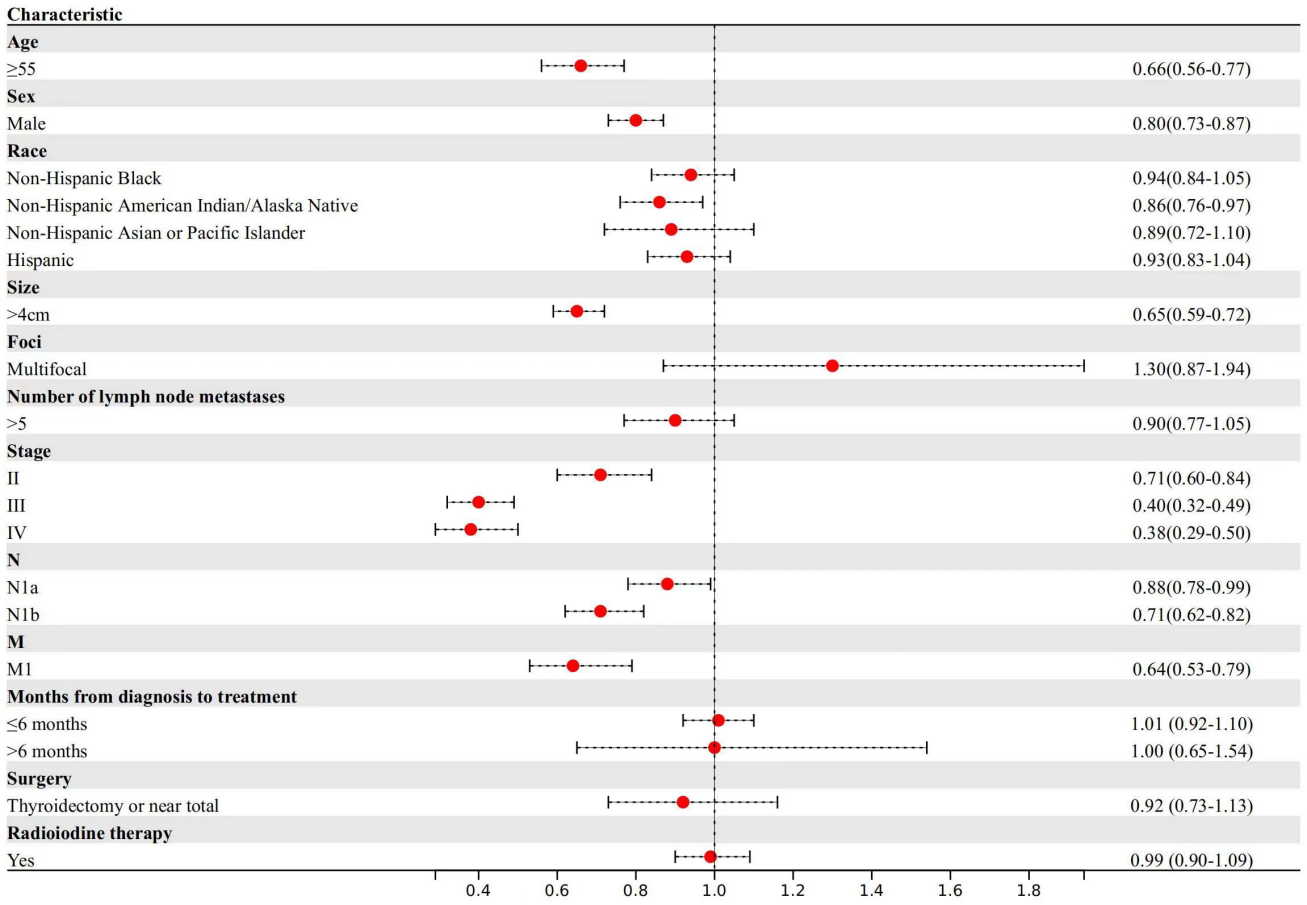
Factors associated with DTC-specific mortality.

Regarding DTC-specific mortality (**Figure 2**), patients aged ≥55 years exhibited a 34% reduction in median survival time compared to those aged <55 years (TR, 0.66; 95% CI, 0.56-0.77). Male patients, in comparison to females, experienced an 20% reduction in median survival time (TR, 0.80; 95% CI, 0.73-0.87). In terms of primary tumor characteristics, patients with larger tumor sizes experienced a 35% reduction in median survival time (TR, 0.65; 95% CI, 0.59-0.72). Similarly, patients with central lymph nodes metastasis and lateral cervical lymph node metastasis had a 12% and 29% reduction in median survival time compared to those without lymph node metastasis, respectively (TR, 0.88; 95% CI, 0.78-0.99; TR, 0.71; 95% CI, 0.62-0.82). Patients with stage II (TR, 0.71; 95% CI, 0.60-0.84) experienced a 23% reduction, those with stage III (TR, 0.40; 95% CI, 0.32-0.49) had a 49% reduction and those with stage IV (TR, 0.38; 95% CI, 0.29-0.50) had a 62% reduction in median survival time for DTC-specific mortality compared to those with stage I disease.

**Figure 3:**
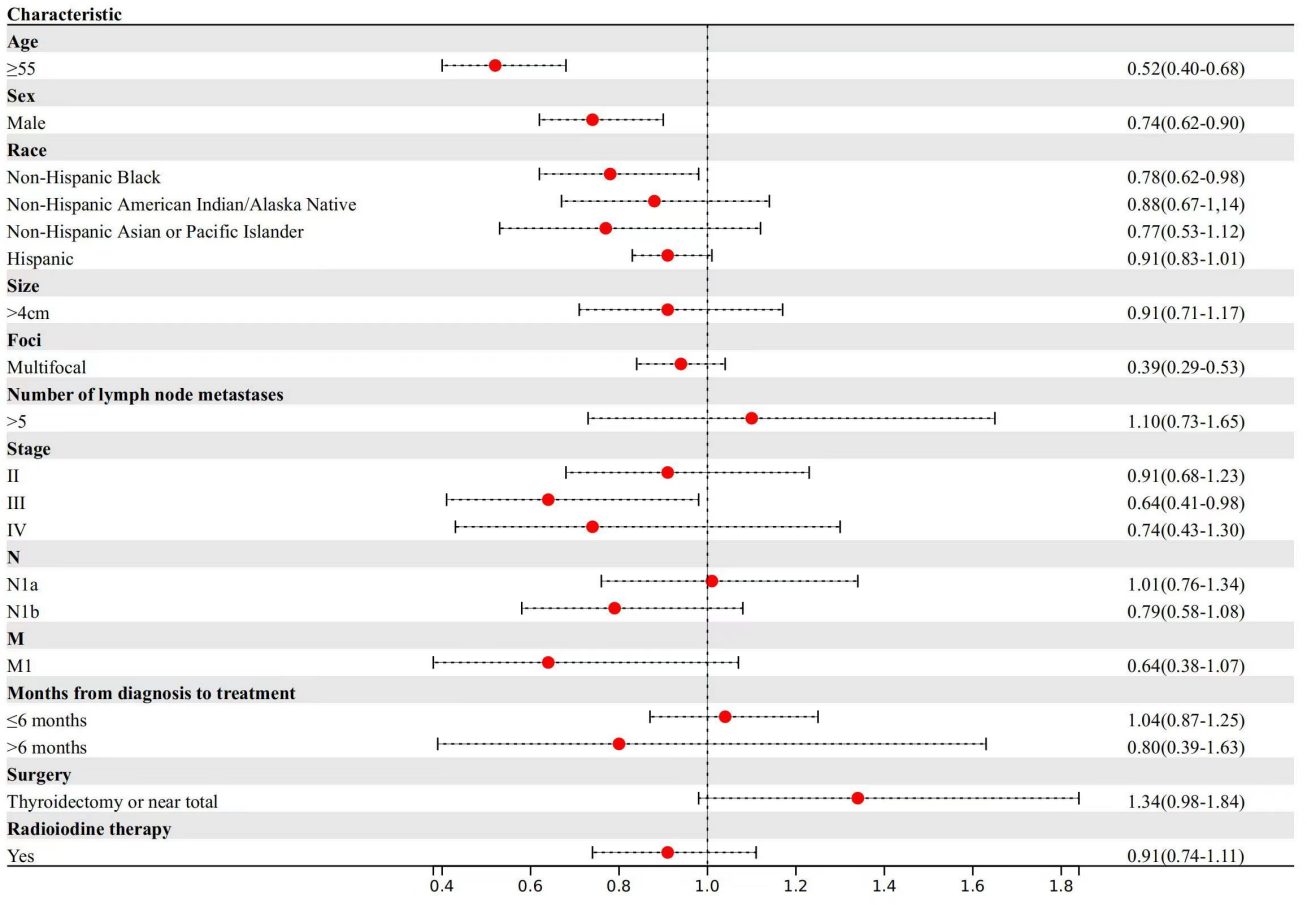
Factors associated with other cancer-specific mortality.

For other cancer-specific mortality (**Figure 3**), patients aged ≥55 years experienced a 48% reduction in median survival time compared to patients aged <55 years (TR, 0.52; 95% CI, 0.40-0.68). Male patients demonstrated a 14% reduction in median survival time (TR, 0.74; 95% CI, 0.62-0.90).

**Figure 4:**
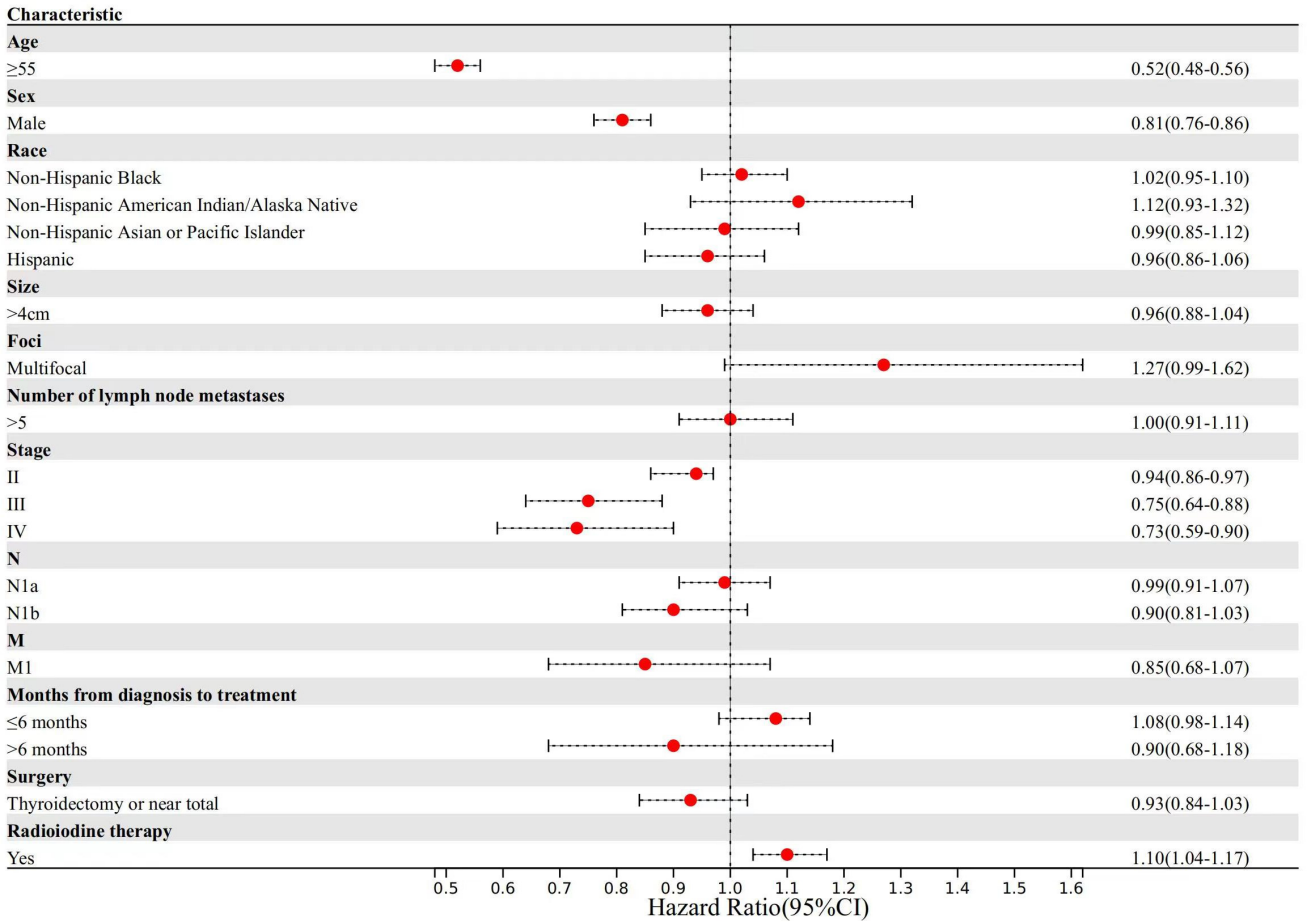
Factors associated with noncancer-specific mortality.

For noncancer-specific mortality (**Figure 4**), patients aged ≥55 years experienced a 48% reduction in median survival time compared to patients aged <55 years (TR, 0.52; 95% CI, 0.48-0.56). Male patients demonstrated a 19% reduction in median survival time (TR, 0.81; 95% CI, 0.76-0.86). Regarding the 8th AJCC TNM staging system, patients in stage II (TR, 0.94; 95% CI, 0.86-0.97) demonstrated a 6% reduction, those in stage III (TR, 0.75; 95% CI, 0.64-0.88) experienced a 25% reduction, and those in stage IV (TR, 0.73; 95% CI, 0.59-0.90) had a 27% reduction in median survival time for noncancer-specific mortality compared to those with stage I disease. Conversely, patients with radioiodine therapy experienced a 10% increase in median survival time (TR, 1.10; 95% CI, 1.04-1.17).

### DTC vs nonDTC-specific cumulative mortality for the entire cohort

**Figure 5:**
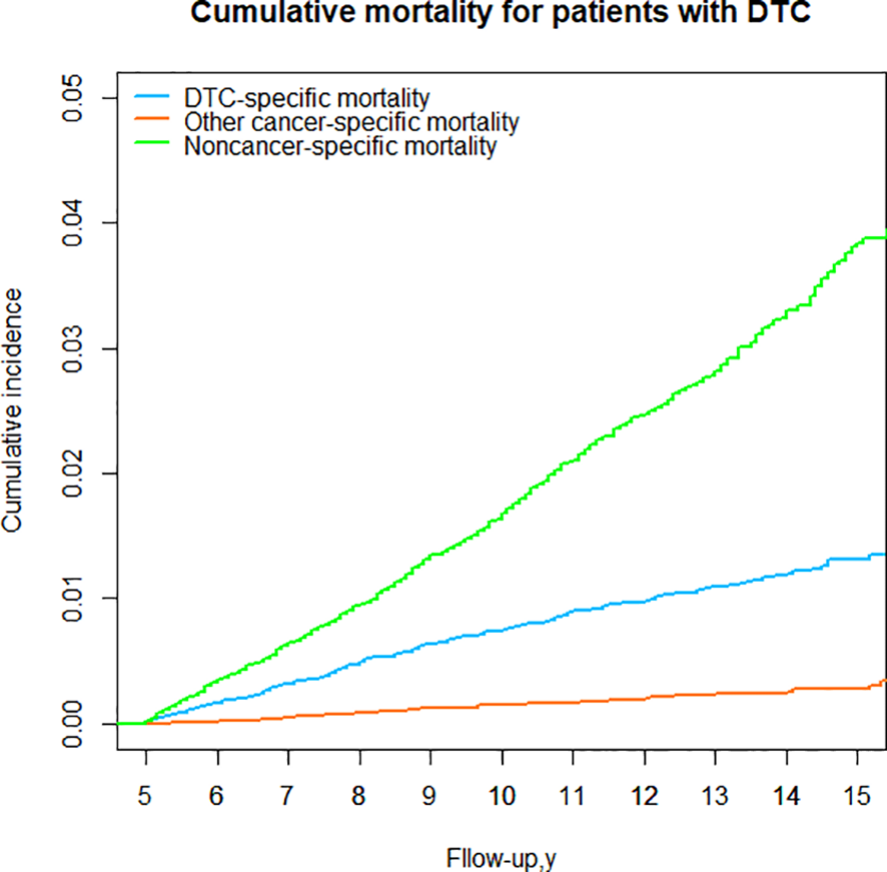
Cumulative mortality for patients with DTC for the entire cohort.

For DTC patients who survived 5 years, a consistent observation was that the cumulative mortality due to noncancer specific causes surpassed that of DTC-specific mortality over the 15-year follow-up period. For the entire cohort, the cumulative incidence of noncancer-specific mortality was 2.93 times higher than the cumulative incidence of DTC-specific mortality from the time of initial diagnosis. However, the cumulative incidence of other cancers was the lowest (**Table 3**; **Figure 5**).

**Table 3:** DTC vs nonDTC-specific cumulative mortality after 15 years of initial diagnosis.

Group	Cumulative incidence of DTC specific mortality (%)	Cumulative incidence of other cancer specific mortality (%)	Cumulative mortality ratio (other cancer vs DTC-specific mortality)	P value	Cumulative incidence of noncancer specific mortality (%)	Cumulative mortality ratio (noncancer vs DTC-specific mortality)	P value
Entire cohort	1.31	0.27	0.21	<0.001	3.84	2.93	<0.001
Sex							
Female	0.80	0.20	0.25	<0.001	3.27	4.08	<0.001
Male	3.24	0.55	0.17	<0.001	6.01	1.86	<0.001
Age							
<55	0.41	0.09	0.21	<0.001	1.40	3.37	<0.001
≥55	4.37	0.91	0.21	<0.001	12.51	2.86	<0.001
Stage							
I	0.37	0.17	0.47	<0.001	2.73	7.44	<0.001
II	6.63	0.84	0.13	<0.001	14.22	2.15	<0.001
III	19.11	2.79	0.15	<0.001	17.11	0.90	<0.001
IV	35.85	2.56	0.07	<0.001	15.57	0.43	<0.001

### DTC vs nonDTC-specific cumulative mortality by age group

**Figure 6:**
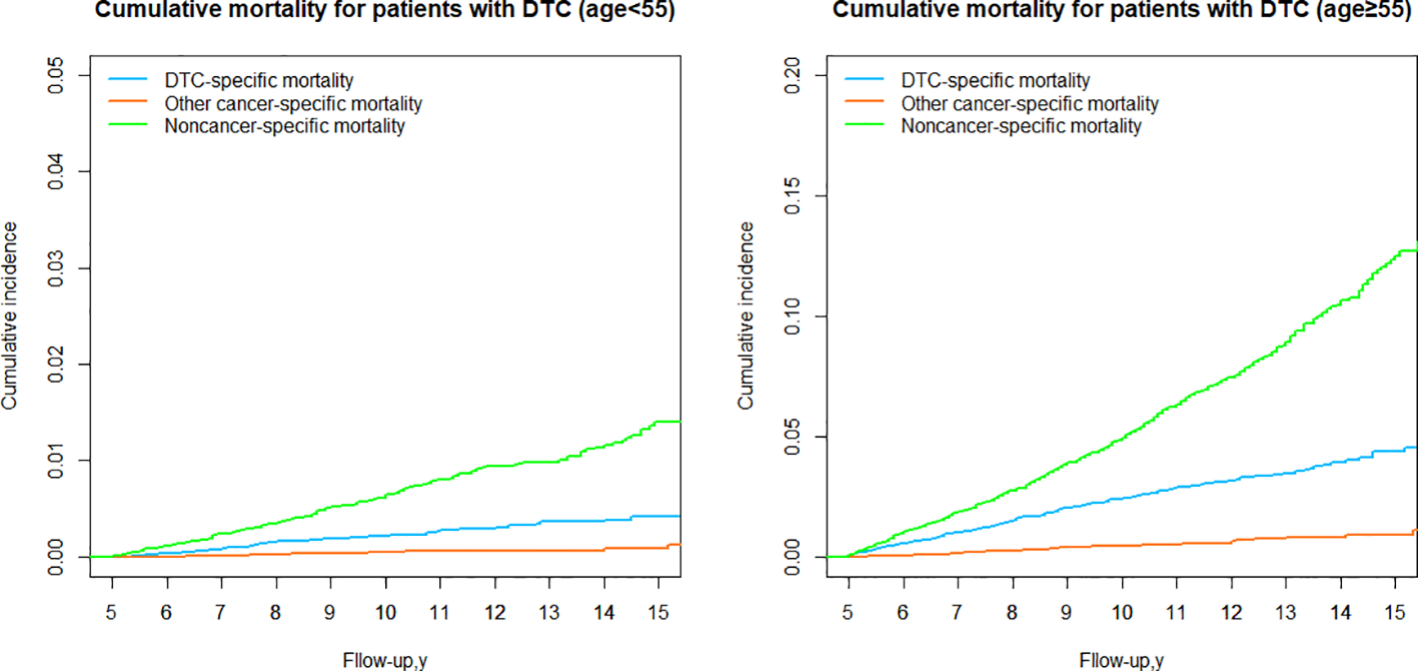
Cumulative mortality for patients with DTC by age group.

We next stratified the patients into two groups based on the 8th AJCC TNM staging system, using a cut-off age of 55 years. In both age groups (younger and older than 55 years), the cumulative mortality due to noncancer-specific causes consistently exceeded that of DTC-specific mortality. However, the cumulative mortality from other cancer-specific causes was consistently lower than DTC-specific mortality. In the group aged <55 years, the cumulative incidence of noncancer-specific mortality was 3.37 times higher than the cumulative incidence of DTC-specific mortality (**Table 3**; **Figure 6**). In the group aged ≥55 years, the cumulative incidence of nonDTC-specific mortality was even more pronounced, being 12.51 times higher than DTC-specific mortality (**Table 3**; **Figure 6**).

### DTC vs nonDTC-specific cumulative mortality by sex group

**Figure 7:**
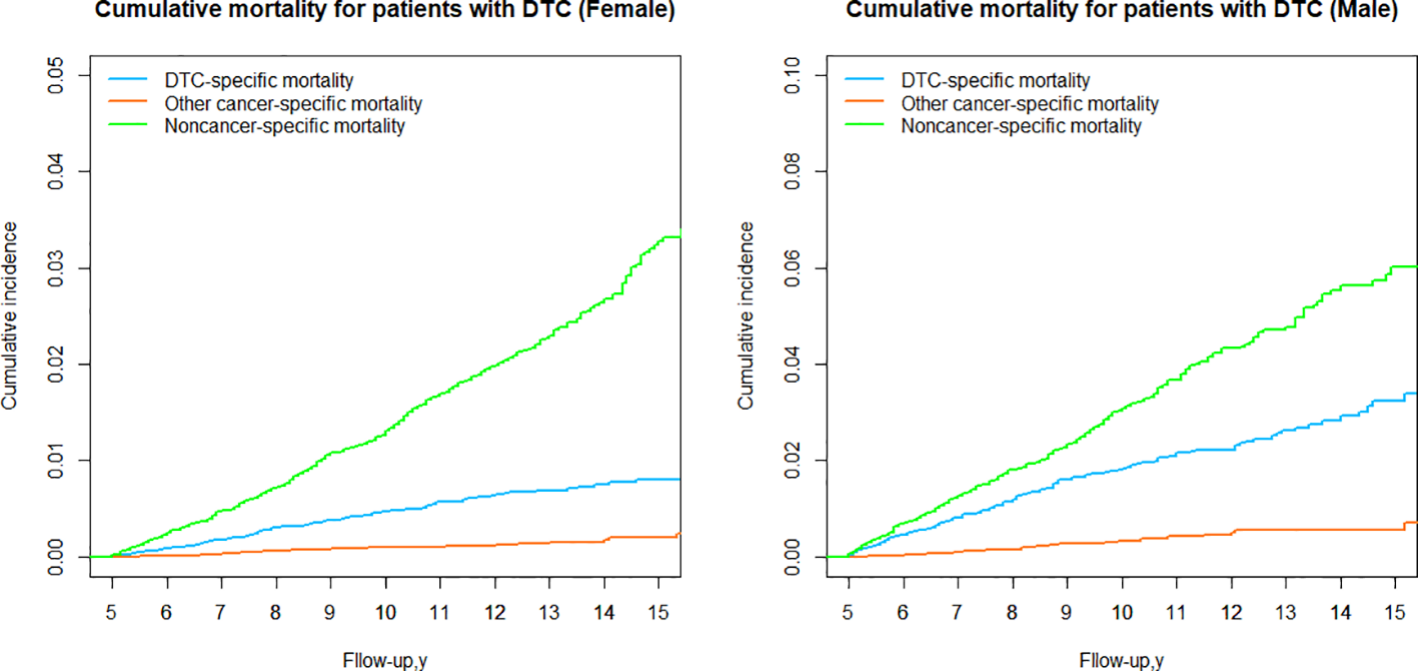
Cumulative mortality for patients with DTC by sex group.

When stratified by sex, it was observed that noncancer-specific cumulative mortality was significantly higher than DTC-specific cumulative mortality in both the female and male (**Table 3**; **Figure 7**) groups, and the cumulative mortality from other cancer-specific causes was consistently the lowest, suggesting that the higher cumulative mortality due to nonDTC-specific causes, compared to DTC-specific mortality, was consistent across genders.

### DTC vs nonDTC-specific cumulative mortality by TNM stage

**Figure 8:**
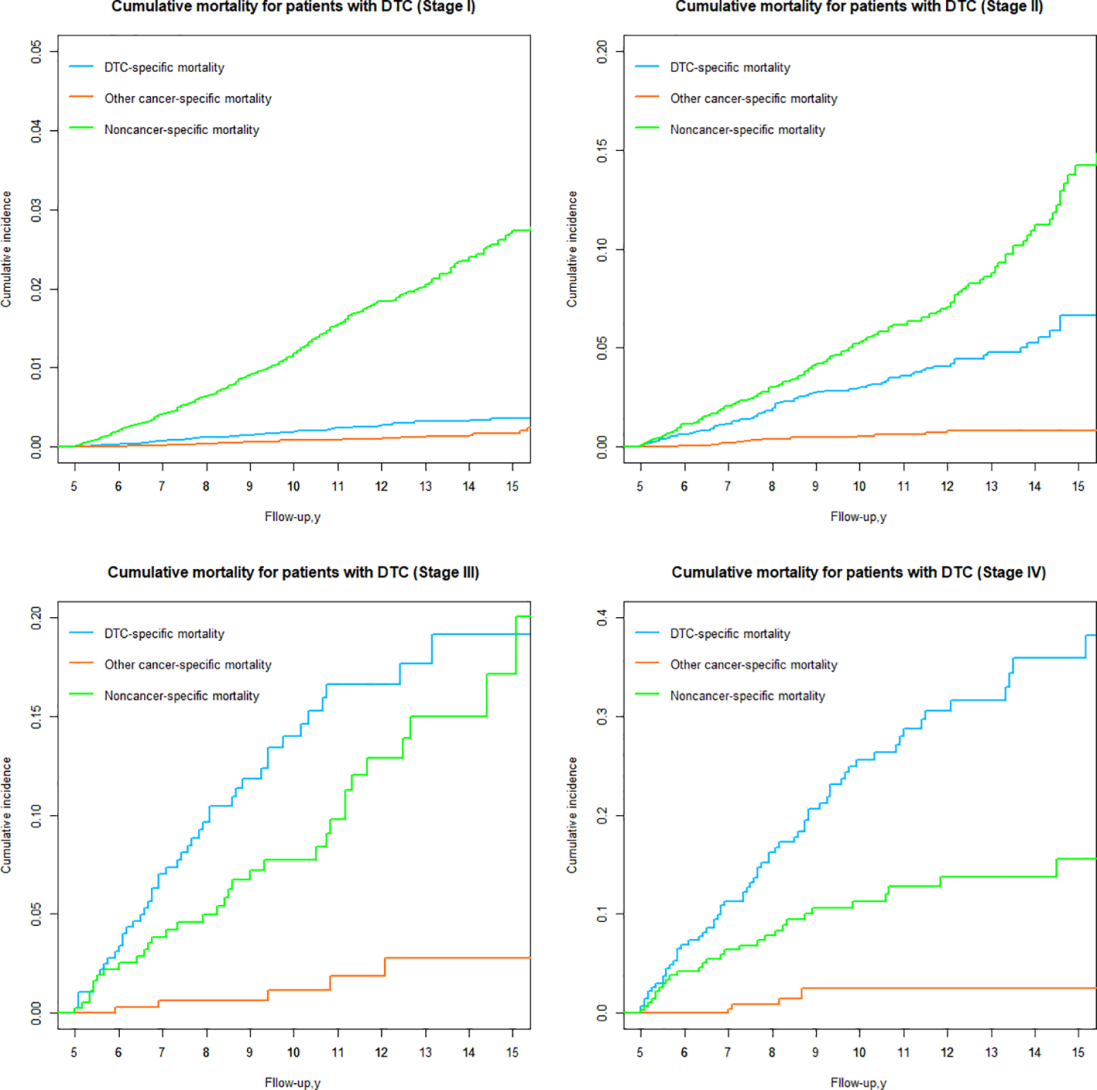
Cumulative mortality for patients with DTC by TNM group.

Based on the 8th AJCC TNM staging system, patients were categorized into four groups (Stages I, II, III and IV), and the risk of DTC versus nonDTC-specific cumulative mortality 15 years after diagnosis varied significantly among all groups (**Table 3**; **Figure 8**).

In stage I, the cumulative incidence of noncancer-specific mortality was nearly 7.44 times higher than that of DTC-specific mortality. Similarly, for patients with stage II (**Table 3**; **Figure 8**), the cumulative incidence of noncancer-specific mortality was almost 2.15 times higher than that of DTC-specific mortality. Among patients with stages I and II, heart disease emerged as the leading cause of noncancer-specific death. For patients with stage III and IV, the cumulative incidence of DTC-specific mortality is the highest of all causes of death, with the cumulative mortality after 15 years of initial diagnosis of 19.11% and 35.85%, respectively. Notably, cardiac disease remained the leading cause of noncancer-specific death for patients with stage III and IV.

The cumulative incidence of other cancer-specific mortality was consistently the lowest among all causes of death for DTC patients with different stages. However, for DTC patients with stage III and IV, the risk of death from other cancers was significantly higher than for patients with stage I and II.

## Discussion

In the realm of thyroid research (4–6), little emphasis has been placed on the relative risk in long-term DTC survivors. Previous research on competing risk models for thyroid cancer (4–7) has primarily utilized histograms to construct predictive models with distinct outcomes. However, these studies did not specifically focus on long-term surviving DTC patients.

In our cohort of long-term survivors, we observed that older age, male gender, size >4cm, presence of distant metastases and the presence of lymph node metastasis were associated with lower cancer-specific survival. This finding aligns with previous studies, all of which have consistently demonstrated an increased risk of disease-specific mortality (4–9). Regarding factors associated with nonthyroid cancer-specific mortality, Yang et al. (6) found that older age, male gender, larger primary tumor, extraglandular invasion, and lack of radioiodine therapy were linked to the cumulative incidence of death resulting from other cancers and noncancer causes. Similarly, our study identified factors associated with nonDTC-specific mortality, including age ≥55 years, male gender, higher stage, and absence of radioiodine therapy, all contributing to a reduction in median survival time.

It is widely thought that estimating the relative risk of cancer versus noncancer-specific mortality in long-term cancer survivors is a crucial step in tailoring specific treatment strategies. Over the years, numerous studies (4–6, 10–17) have explored cancer and noncancer-specific risks for various common cancers such as breast, prostate, and colorectal cancers. Cai et al. (16) found differences in colon cancer-specific mortality (CCSM) and non-colon cancer-specific mortality (NCCSM) based on age groups among patients with stage I and II colon cancer (P<0.001). The study noted that patients under 50 years of age exhibited more aggressive features but lower CCSM, while older patients faced a significantly higher risk of both CCSM and NCCSM. In the present study, we employed AFT modeling to identify factors associated with DTC and nonDTC-specific mortality in long-term survivors of papillary thyroid cancer (PTC), considering competing events. Moreover, we found that both DTC and nonDTC-specific mortality were higher in older and male patients compared to younger and female patients, with noncancer-specific mortality consistently surpassing DTC-specific mortality. Further comparisons based on risk groups in breast cancer and prostate cancer studies revealed noteworthy findings. For low-risk breast cancer patients, the cumulative incidence of non-breast cancer-specific mortality (NBCSM) was nearly seven times higher than the cumulative incidence of breast cancer-specific mortality (18). Rasul et al. (17) reported that intermediate and high-risk prostate cancer patients exhibited a greater likelihood of mortality from alternative malignancies, cardiovascular disorders, and additional causes of death compared to those with low-risk prostate cancer. Similarly, a study based on the SEER database revealed that the cumulative incidence of nonprostate cancer-specific mortality in the low-risk group was nearly nine times higher than the cumulative incidence of prostate-specific mortality (18). Madhav et al. (18) observed a 40% reduction in median survival time for CCSM and an 8% reduction in median survival time for NCSM in patients with stage III colon cancer compared with stage I. The median survival time for NCSM in the low-risk colon cancer group was seven times greater than the median survival time for CCSM in the low-risk colon cancer cohorts. Similar trends were observed for stage I and stage II. For patients with stage III and IV, the cumulative mortality from primary tumors far exceeds that from other diseases. Consequently, thyroid-related prognosis should be given extra consideration. After this period, as the cumulative mortality from other diseases increases, extra attention should be directed toward diseases other than the primary tumor, especially diseases of the circulatory system. Collectively, these findings suggest that, for common malignancies, the risk of noncancer mortality is notably higher than the risk of cancer mortality in patients with stage I and II or low-risk diseases. Conversely, for stage III and IV patients, the risk of mortality from primary cancer becomes more prominent. This observation may be attributed to the relatively low malignancy of DTC, which reduced the risk of death with increasing survival time, leading to the risk of noncancer death becoming higher than the risk of cancer death at some time postoperatively.

Cardiovascular diseases are a leading cause of death worldwide. According to data from the World Health Organization, in 2019, the number of deaths due to cardiovascular diseases exceeded 15 million, accounting for 27% of total global deaths (19). In a study involving 7622 adults aged ≥20 years in the United States, there were 532 recorded deaths during a median follow-up of 5.8 years, with 186 of those deaths attributed to cardiovascular diseases (20). According to estimates from the Global Burden of Disease (GBD), cardiovascular diseases caused 388268 deaths in Brazil in 2017, accounting for 27.3% of the total mortality in the country (21). According to the 2017 GBD data, the majority of deaths were caused by ischemic heart diseases, accounting for 175,791 deaths (30%) (21). Oral sodiumlevothyroxine is routinely required for TSH suppression therapy in DTC patients after surgery. Some studies have indicated that sodium levothyroxine increases the risk of adverse effects, including cardiac complications (arrhythmias, atrial fibrillation, angina pectoris, myocardial infarction) and skeletal complications (osteoporosis, osteopenia, fractures) (22–24). Additionally, studies have shown that low TSH levels are associated with an increased risk of cardiovascular mortality and atrial fibrillation (25). In long-term survivors of DTC, the mortality rate due to cardiovascular diseases exceeds 30%. We speculate that the increased risk of cardiovascular mortality in DTC patients is associated with long-term TSH suppression.

In the study by Yang et al. (6), the 10-year cumulative incidence of death resulting from noncancer causes/thyroid cancer in PTC patients was 1.59 (3.5%/2.2%), and 1.33 (6.4%/4.8%) for follicular thyroid carcinoma (FTC). In our long-term survival DTC patients, the ratio was 2.93 (3.84%/1.31%). For the 10-year cumulative incidence of death resulting from other cancer causes/thyroid cancer in PTC patients was 0.86 (1.9%/2.2%), and 0.50 (2.4%/4.8%) for follicular thyroid carcinoma (FTC). In our long-term survival DTC patients, the ratio was 0.21 (0.27%/1.31%). This suggests that for patients with DTC, the risk of death from non-cancer diseases gradually dominates as survival time increases, whereas the risk of death from other cancers gradually decreases. Simultaneously, it emphasizes the crucial importance of dynamically observing the prognosis of patients with DTC.

### Limitation

The findings of this study should be interpreted in light of certain limitations. Firstly, the SEER database lacks information regarding the comorbidities of patients with other diseases. Secondly, the utilization of the SEER database, limited to the U.S. population, may restrict the generalizability of the results to other populations. Thirdly, the inherent limitations of registry data, such as potential issues with coding inaccuracies or missing data, might have affected the precision of variable capture. Fourthly, the absence of information on the duration, dose, or frequency of postoperative adjuvant therapy hindered our ability to interpret the impact of radiation therapy on survival. Fifthly, due to the lack of relevant data, we were unable to analyze recurrence-free survival rates.

## Conclusion

The observed variation in the risk of DTC versus nonDTC-specific mortality across different cancer stages emphasizes the importance of implementing personalized, risk-stratified treatment strategies. Specifically, DTC patients diagnosed with stage III and IV should receive heightened attention to the primary cancer in the early postoperative period. Conversely, for DTC patients with stage I and II, while maintaining focus on thyroid-related concerns, extra attention should be directed towards assessing the impact of other diseases, especially cardiac disease. Indeed, comprehending the relative burden of cancer and noncancer mortality among long-term survivors of DTC is crucial for developing more tailored and effective follow-up protocols.

## Authors’ note

The authors hereby confirm that neither the manuscript nor any part of it has been published or is being considered for publication elsewhere. We acknowledge that all authors participated sufficiently in the work and take public responsibility for its content. This article strictly follows the STROBE statement.

## Data Availability

The original contributions presented in the study are included in the article/supplementary material. Further inquiries can be directed to the corresponding authors.
